# Revealing the Best Strategies for Rare Cell Type Detection in Multi-Sample Single-Cell Datasets

**DOI:** 10.3390/genes17010031

**Published:** 2025-12-29

**Authors:** Zhiwei Ye, Yinqiao Yan, Yuanyuan Yu, Hao Wu

**Affiliations:** 1 Department of Electronic and Electrical Engineering, Southern University of Science and Technology, Shenzhen 518055, China; 12333442@mail.sustech.edu.cn; 2 Faculty of Computer Science and Control Engineering, Shenzhen University of Advanced Technology, Shenzhen 518107, China; 3 School of Mathematics, Statistics and Mechanics, Beijing University of Technology, Beijing 100124, China; yinqiaoyan@bjut.edu.cn; 4 Institute of Advanced Computing and Digital Engineering, Shenzhen Institute of Advanced Technology, Chinese Academy of Sciences, Shenzhen 518055, China

**Keywords:** rare cell detection, single-cell RNA sequencing (scRNA-seq), population-level analysis

## Abstract

**Background**: Single-cell RNA sequencing (scRNA-seq) enables high-resolution characterization of cellular heterogeneity and provides unique opportunities to identify rare cell populations that may be obscured in bulk transcriptomic data. However, despite the growing interest in rare-cell discovery, most existing detection methods were originally developed for single-sample datasets, and their behavior in multi-sample settings—where batch effects, sample imbalance, and heterogeneous cell-type compositions are common—remains poorly understood. This study aims to systematically evaluate representative rare cell detection methods under multi-sample settings and identify the most effective analytical strategies. **Methods**: We performed a comprehensive benchmarking analysis of five widely used rare cell detection tools, CellSIUS, GapClust, GiniClust, scCAD, SCISSORS and a scGPT-based rare cell detection method using Isolation Forest. Each method was evaluated under three analytical strategies: individual sample detection, pooled sample detection, and batch-corrected pooled sample detection. Performance was assessed across multiple publicly available scRNA-seq datasets using standardized evaluation metrics. **Results**: Batch-corrected pooled sample detection consistently achieved the highest overall performance across methods and datasets, whereas individual sample detection produced the weakest results. Among the evaluated tools, scCAD demonstrated the most robust and stable performance across dataset types and analytical conditions. **Conclusions**: This study provides strategy-level comparison in multi-sample settings. Our findings highlight the importance of batch correction and pooled analysis for improving rare cell detection accuracy and offer practical guidance for selecting optimal methods and analytical workflows in large-scale single-cell transcriptomic studies.

## 1. Introduction

Single-cell RNA sequencing (scRNA-seq) is a powerful technology for investigating complex biological systems [1,2]. By measuring gene expression at single-cell resolution, it enables researchers to dissect cellular heterogeneity with very fine detail and to characterize diverse cell types, states, and dynamic regulatory processes within complex tissues [3,4]. Beyond identifying major cell populations and performing clustering analysis, scRNA-seq also makes it possible to detect rare cell populations that occupy a very small fraction of the dataset and are otherwise difficult to capture [5,6,7], yet these cells often have substantial biological and clinical relevance, playing critical roles in tissue development, disease progression, and immune response [3,8,9]. Examples include stem cells that maintain tissue homeostasis, early progenitors that guide developmental trajectories, and circulating tumor cells that seed metastasis [10,11]. Therefore, detecting rare cells is of critical importance in developmental biology, cancer research, and precision medicine [12,13]. However, the inherent sparsity and technical noise in single-cell datasets pose significant challenges for rare cell detection, especially when the target population constitutes less than 1% of the total cells [14,15].

In recent years, a number of computational methods have been developed specifically for rare cell detection, including clustering-based methods that leverage structural differences (e.g., GapClust (v0. 1. 0) [16]), outlier detection frameworks (e.g., scCAD [17]), and hybrid methods that combine clustering with gene expression modeling (e.g., CellSIUS [18], SCISSORS [19], GiniClust [20,21]). We will briefly review these methods in a later section. However, most of these methods were designed primarily for data generated from a single sample and do not explicitly account for inter-sample variation. For example, some rare cell types may be present in a large fraction of samples, whereas others appear only sporadically, leading to considerably different signal strengths at the population level. Recently, large-scale population-level scRNA-seq studies have become a key resource for capturing cell-type-specific biological signals across diverse samples [22,23,24]. They enable the identification of disease-associated cell states, genetic and environmental influences on cell function, and more robust biomarkers for translation into clinical practice [22,23,24]. For these data, the question of how rare cell types should be defined and called is particularly intriguing and warrants further investigation.

For multi-sample scRNA-seq data, if a cell type is flagged as “rare” but appears in only a very small number of subjects, then it is reasonable to suspect that such a cell population may reflect technical artifacts or sample-specific events [25,26]. Only when a rare cell type is consistently detected across multiple samples can it be confidently regarded as a true rare population [27]. Therefore, population-level studies not only enhance the robustness of rare cell detection but also provide essential evidence to distinguish genuine rare cell types from sample-specific noise [28,29]. In multi-sample scRNA-seq settings, the rare cell type detection becomes more complicated because a rare cell type requires a definition that explicitly accounts for multiple samples and their heterogeneity [30,31]. Loosely speaking, a rare cell type in population-level scRNA-seq should satisfy (1) in one subject, there are multiple cells rather tightly clustered; (2) this cell type is rather far away from abundant cell types; and (3) cells of this type appear in multiple subjects. The first two criteria define the signal-to-noise ratio (SNR) and are also the basis in single subject rare cell type detection, and the third criterion introduces a population-level dimension that must be considered jointly with the other two in order to make rare cell calls biologically credible [16,21].

For rare cell type detection from multi-sample scRNA-seq data, researchers can adopt three analytical strategies. The first is the individual sample detection strategy, in which rare cells are identified independently within each sample. The second is the pooled-level detection strategy, where data (expression matrices) from multiple samples are merged and analyzed jointly. The third strategy is batch-corrected pooled detection, where data from different individuals are first harmonized to reduce inter-sample technical variation before joint analysis [32,33,34]. These strategies could lead to markedly different results and can substantially affect the sensitivity, robustness, and accuracy of rare cell detection results. Each strategy is based on distinct assumptions and offers unique strengths and weaknesses. Specifically, the individual detection strategy assumes that rare cells can be identified within a single subject. It emphasizes preserving subject-specific information and avoids potential confounding introduced by data integration [6,8]. Its strength lies in detecting individual-specific rare populations and enabling condition-specific comparisons (e.g., disease vs. control). However, the limited number of rare cells per sample often results in reduced statistical power and unstable detection. The population-level detection strategy assumes that rare populations are reproducibly present across multiple individuals. By pooling data, it increases the absolute number of rare cells, thus improving statistical power and recall [35]. Its advantage is the ability to better distinguish true rare populations from random noise, but it may obscure subject-specific patterns and is more vulnerable to inter-sample heterogeneity [29]. Finally, in the batch-corrected pooled detection strategy, batch-effect correction is first applied to remove inter-sample technical variation, after which rare cell detection is performed jointly across multiple samples. This preprocessing step aligns shared biological signals across individuals while minimizing technical noise, thereby improving cross-sample comparability [26,36,37]. Nevertheless, excessive correction may risk removing subtle but biologically meaningful differences.

Despite the growing use of these three strategies, it is still unclear which would be the most appropriate strategy in multi-sample scRNA-seq rare cell type detection. In this work, we present a comprehensive comparison of five state-of-the-art rare cell detection methods under all three strategies. Through extensive simulations and real-data analyses, we quantify each method’s accuracy, stability, and cross-sample consistency, providing practical guidance on when and how to apply these strategies for reliable rare cell discovery in multi-sample single-cell studies.

## 2. Materials and Methods

### 2.1. Methods Under Comparison

We systematically collected published rare cell detection methods by searching the literature using the keywords “rare cell detection” and “single-cell RNA sequencing” (scRNA-seq). Among all these methods, we ultimately selected five for benchmarking because they (1) are publicly available with accessible source code, (2) can be installed and executed successfully in our computing environment, (3) operate on standard single-cell inputs (expression matrix and cell annotations) without requiring additional modalities such as spatial coordinates or protein measurements, (4) do not depend on paired disease/control designs, and (5) allow users to specify or control the target rare cell proportion (Table 1).

These six methods span diverse algorithmic principles and computational frameworks when performing rare cell detection. Specifically, CellSIUS identifies rare subpopulations by detecting bimodal gene expression patterns within clusters and constructing co-expression modules for refined subclustering [18]. GapClust is a fully unsupervised clustering algorithm that is based on local density differences and large “gaps” between core and peripheral cells in high-dimensional expression space [16]. GiniClust selects high-Gini genes that are expressed in only a few cells and performs clustering in a reduced-dimensional space to detect rare populations [20]. scCAD frames rare-cell detection as a cluster-level anomaly task: it first performs feature selection (e.g., HVGs/RFGs), then decomposes cells into many balanced sub-clusters, merges highly similar sub-clusters, and finally computes anomaly scores per sub-clusters, assigning rare labels to the cells in clusters that are both small and show high independence [17]. ScGPT + isolation forest (IF) represents an exploratory method in which we leverage a single-cell foundation model [38,39]. We first use the pretrained scGPT model to transform each cell’s gene expression profile into a latent embedding, and then apply Isolation Forest to these embeddings to compute anomaly (rare-cell) scores. Cells are subsequently ranked by their anomaly scores, and those with the highest scores are designated as rare cells. SCISSORS refines existing clusters by evaluating the silhouette scores of individual cells, identifying loosely embedded outliers, and re-clustering them at higher resolution to reveal potential rare subpopulations [19].

All methods were executed using their recommended or default settings and were evaluated under three analytical paradigms: (1) individual detection, where rare cells were identified independently within each sample; (2) pooled detection, in which multiple samples were merged and analyzed jointly; and (3) batch-corrected pooled detection with batch correction, where data were first integrated using ComBat-seq to mitigate batch effects prior to detection [40]. For all methods, parameters were set to recommended or empirically balanced values to ensure comparable detection sensitivity. In CellSIUS (v1. 0. 0), min_n_cells = 3, min_fc = 1, and corr_cutoff = 0.7 controlled the minimal cluster size, within-cluster fold change, and gene–gene correlation threshold. In GapClust (v0. 1. 0), the neighbor number k was set to ~1% of total cells, with distance = “euclidean” and minPts = 5 to define local density gaps. In GiniClust3 (v1. 1. 2), min_gini_value = 0.3, neighbors = 10, and minPts = 5 were used to select high-Gini genes and small clusters. In scCAD (implemented from the official GitHub repository https://github.com/xuyp-csu/scCAD), merge_h = 0.3, overlap_h = 0.7, and dataset-dependent rare_h controlled cluster merging and rarity thresholding. In SCISSORS (v1. 2. 0), k.vals = (20, 30, 40), resolution.vals = (0.2, 0.3, 0.4), n.PC = 20, and n.HVG = 2000 guided iterative reclustering. For scGPT (v0. 2. 4) + Isolation Forest, embeddings were generated with batch_size = 256, and outliers were detected using n_estimators = 500 and rare_fraction = 0.01. In addition, we have written open and reproducible Jupyter notebooks with tutorials for all the available tools, which can be downloaded from our website [41].

As shown by the UMAP visualizations before and after ComBat-seq correction (Supplementary Figures S1–S4a), cells of the same biological type from different samples are clearly separated prior to integration, whereas after correction, they cluster together across samples. Importantly, major cell types remain well separated and biologically interpretable after integration, indicating that batch correction does not induce artificial merging or loss of structure. Furthermore, canonical marker genes for rare cell types, such as *IL3RA* and *CLEC4C* for pDC, *TPSAB1* for mast, and established neuronal subtype markers, remain specifically and strongly expressed after batch correction.

### 2.2. Dataset and Gold Standard

We applied all five methods to four publicly available scRNA-seq datasets that include multiple subjects, which exhibit inter-individual variability in both the compositions and proportions of rare cell types. The datasets used in this study are listed in Table 2, including the following biological tissues: human breast cancer, human midbrain, human kidney, and mouse hippocampus, each comprising approximately ten samples [42,43,44,45]. These datasets differ in biological context, tissue composition, and sample characteristics, allowing us to evaluate method performance under diverse real-world conditions.

There are three conditions that can influence the performance of rare cell detection: (1) Number of rare cell types: some datasets contain a single rare population, whereas others include multiple distinct rare subgroups; (2) Intra-sample heterogeneity: the degree of variation across samples differs among datasets, encompassing patient-to-patient variability and tissue-specific expression patterns; (3) Rare cell proportion: the abundance of rare cells ranges from <0.5% to ~1.5%, thereby representing both ultra-rare and moderately rare detection scenarios. Taken together, the four selected datasets cover the scenarios described above and thus provide a solid basis for evaluating detection performance across diverse biological contexts.

We utilized the following scheme to determine the true rare cell types in each real dataset based on annotated cell types and their abundance. From the perspective of discovering rare cell types, ideal rare cells are expected to be both low in proportion and distinct in gene expression from major cell populations. However, in practice, defining such expression differences is ambiguous and depends heavily on annotation quality. Therefore, we adopted a quantitative criterion: only datasets with known cell-type annotations were used, and cell types accounting for less than a predefined threshold (e.g., 1% of total cells) within each sample were labeled as rare. To further support the biological validity of these annotated rare populations, we additionally examined the expression of canonical marker genes reported in the datasets (Supplementary Figures S1–S4). Across all datasets, the low-abundance populations exhibited clear enrichment of their known markers (e.g., *IL3RA*, *CLEC4C* for pDC; *TPSAB1* for Mast; *Hbb-bt*, *Hbb-bs* for Blood), confirming that these annotated rare cell types represent biologically meaningful cell states rather than technical artifacts. These validated rare populations were used as the ground truth for computing true and false positives across detection methods. These low-abundance annotated cell types were regarded as true rare cells (true positives) and were used to compute true positives and false positives across methods, forming a consistent and reproducible benchmark reference.

In addition to real datasets, we generated simulated datasets as a supplementary validation tool to evaluate the stability and reproducibility of the methods under controlled conditions. While real data capture complex biological variability, they lack a carefully designed ground truth, making it difficult to assess consistency across repeated runs or varying data properties. To address this, we constructed simulated datasets by resampling and adjusting the Breast Cancer datasets to meet predefined parameters. Two parameters were systematically adjusted: (1) rare cell proportion (0.5–1.5%), and (2) number of rare cell types (1–3). These simulations were not intended for strategy comparison, but rather to test each method’s ability to discover the true rare populations under extremely weak signals.

These validated rare populations were used as the ground truth for computing true and false positives across detection methods. We note that this definition represents an operational rather than biological gold standard. Although low-abundance annotated populations provide a reproducible benchmark reference, they do not capture all biologically meaningful rare states, and their accuracy depends on the quality of the original annotations. Thus, the benchmark ground truth should be interpreted as abundance- and annotation-based, not a definitive biological standard.

### 2.3. Data Preprocessing

As described above, we collected multi-subject scRNA-seq datasets from the Gene Expression Omnibus (GEO, https://www.ncbi.nlm.nih.gov/geo/ (accessed on 26 December 2025)). Each dataset contains both an expression matrix and corresponding cell type annotations. For the Breast Cancer and Human Kidney datasets, we downloaded the processed expression matrices, which already contained normalized gene expression values and cell-type metadata. These data were directly converted into Seurat objects for subsequent analyses. In contrast, the Mouse Hippocampus and Human Midbrain datasets only provided raw count matrices. Therefore, we processed these data following the standard Seurat workflow [46], which included: (1) filtering out low-quality cells (retaining cells with >200 detected genes and <10% mitochondrial gene content); (2) normalization was performed using the NormalizeData function, with joint normalization applied for the pooled detection strategies; (3) identification of highly variable genes and principal component analysis (FindVariableFeatures, RunPCA). Specifically, HVG selection was performed per sample in the individual detection strategy, whereas global HVGs were used for the pooled detection strategies; and (4) construction of nearest-neighbor graphs and clustering (FindNeighbors, FindClusters). All datasets were pre-processed under the same criteria to ensure consistency across different detection methods. For convenience, all data were stored as Seurat objects, with the cell-type information recorded in the meta.data$cell.type field. These Seurat objects were then converted into matrix and metadata files to facilitate compatibility with Python-based tools such as scCAD and GiniClust. The complete pre-processing workflow and code will be made publicly available in our GitHub repository upon publication (https://github.com/arcane-11/rare-cell-detection-benchmark (accessed on 26 December 2025)).

### 2.4. Benchmark Workflow

#### 2.4.1. Run Rare Cell Detection Methods and Collect Results

We ran all five rare cell detection tools on each sample independently, using the authors’ recommended or default parameters. No additional parameter tuning was performed to ensure the fairness and reproducibility of comparisons.

Each method was executed under three analytical strategies—individual detection, pooled detection, and batch-corrected pooled detection—using the same datasets. For pooled detection, cell-level normalization and feature selection were performed before sample integration. For batch-corrected pooled detection, considering that all samples subjected to batch correction originated from the same dataset and therefore exhibited no substantial differences in sequencing depth across cells, batch effects were corrected using ComBat prior to detection [47]. All analyses were based on cell-by-gene expression matrices as input, without incorporating additional information such as cell-type labels.

To ensure consistency across tools, we applied a unified workflow to collect and process outputs as follows:

Detection Execution: Each method was executed through its complete workflow to obtain predicted results, including rare cell indices or labels.

Format Standardization: Outputs from different algorithms—such as subcluster labels, outlier scores, or binary rare cell indicators—were converted into a standardized format, where 0 denotes common cells and 1 denotes rare cells.

Result Evaluation: Based on the defined gold standard, we obtained the following numbers: true positive (*TP*) represents correctly identified rare cells, false positive (*FP*) represents common cells incorrectly predicted as rare, true negative (*TN*) represents correctly identified common cells, and false negative (*FN*) represents true rare cells that were missed by the method.

Performance Computation: Using these statistics, we further calculated *Precision*, *Recall* (Sensitivity), Specificity, *F*1-score, Matthews correlation coefficient (*MCC*), Cohen’s κ, and *G-mean* as quantitative performance metrics.

In addition, we evaluated the processing time and memory usage of each tool. For R-based implementations (e.g., CellSIUS, GapClust, and SCISSORS), we recorded runtime using the proc.time() function; for Python-based implementations (e.g., scCAD sc GPT + IF and GiniClust), we measured runtime using the Linux command time. All tools were executed on the same hardware environment to ensure comparability. It is important to note that not all methods successfully ran on every dataset. For cases where a method terminated with errors or failed to produce output, we labeled the result as “error” and assigned a processing time of NA, as shown in Supplementary Table S1. In contrast, if a method completed successfully but detected no rare cells, the output was regarded as “no significant rare population” and its actual processing time was retained. To automate this distinction, we implemented custom script functions to extract both detection status and corresponding runtime from each tool’s log files.

Finally, we summarized and compared each method’s performance, stability, and computational efficiency under the three analytical strategies to provide a comprehensive evaluation and method recommendation framework.

#### 2.4.2. Evaluation

To quantitatively assess the performance of rare cell detection methods, we adopted a set of metrics commonly used in imbalanced binary classification tasks. In our setting, rare cells are treated as the positive class, while non-rare (common) cells are treated as the negative class. Given that rare cells typically account for a very small fraction of the total population, the class imbalance is extreme, making accuracy an inappropriate evaluation metric. Therefore, we selected the following six metrics, each reflecting different aspects of detection performance.

- *Precision* quantifies the proportion of correctly predicted rare cells among all cells predicted as rare. It is particularly informative when controlling for false positives and is defined as:

(1) $$Precision=\frac{TP}{TP+FP}$$

- *Recall* (Sensitivity) measures the proportion of true rare cells correctly identified by the method, reflecting its ability to minimize false negatives. It is computed as:

(2) $$Recall=\frac{TP}{TP+FN}$$

- *F*1-score represents the harmonic mean between *Precision* and *Sensitivity*, indicating the balance between detection accuracy and completeness. It is calculated as:

(3) $$F1=\frac{2TP}{2TP+FP+FN}$$

- Matthews correlation coefficient (*MCC*) provides a balanced measure that considers both true and false predictions across positive and negative classes. It is particularly robust for imbalanced datasets and is defined as:

(4) $$MCC=\frac{TP*TN-FP*FN}{\sqrt{\left(TP+FP)(TP+FN)(TN+FP)(TN+FN\right)}}$$

- *Kappa* (Cohen’s κ) quantifies the level of agreement between the predicted and true labels, correcting for random chance. It is calculated as:

(5) $$Kappa=\frac{p_{o}-p_{e}}{1-p_{e}},\;\mathrm{where}\;p_{o}=\frac{TP+TN}{TP+FP+FN+TN},p_{e}=\frac{\left(TP+FP\right)\left(TP+FN\right)+(TN+FN)(TN+FP)}{{\left(TP+FP+FN+TN\right)}^{2}}$$

- *G-mean* evaluates the geometric mean between Sensitivity and Specificity, reflecting the balance between detecting rare cells and avoiding false positives. It is defined as:

(6) $$G-mean=\sqrt{\frac{TP}{TP+FN}\cdot \frac{TN}{TN+FP}}$$

## 3. Results

### 3.1. Overall Performance Comparison

**Figure 1:**
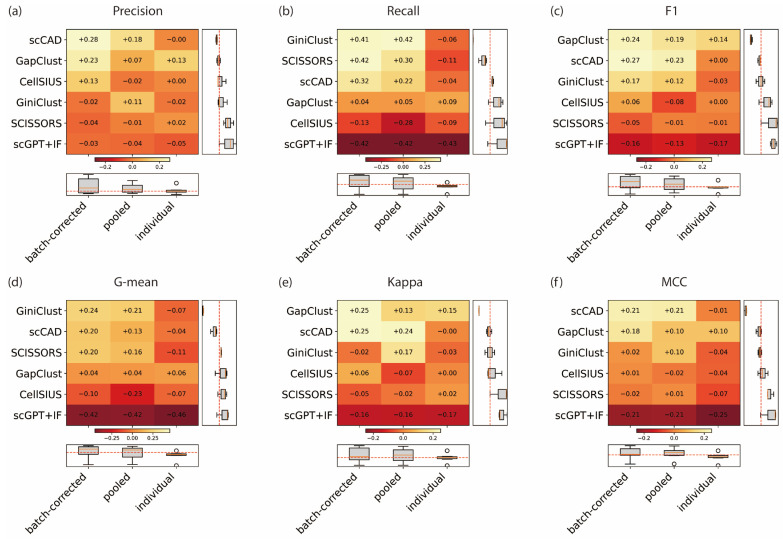
Performance gain/loss of five rare cell detection methods under three analytical approaches across all datasets. (**a**) *Precision*, (**b**) *Recall*, and (**c**) *F*1-score, (**d**) *G-mean*, (**e**) *Kappa*, (**f**) *MCC*. Each heatmap illustrates the relative performance changes (gain or loss) of CellSIUS, GapClust, GiniClust, scCAD, scGPT + IF and SCISSORS under three detection strategies: individual detection, pooled detection, and batch-corrected pooled detection. Values in each cell represent the average performance difference compared with the overall baseline. The heatmaps are arranged by average row and column values, with higher scores (lighter colors) indicating improved performance. Boxplots on the right and bottom display the marginal gain/loss distributions for each method and approach. The red dashed line in each boxplot denotes the zero reference (no gain or loss).

We compared five rare cell detection methods under three strategies across four multi-sample datasets and calculated six metrics for each. In total, we obtained 360 resulting metric values. Since each dataset exhibits different baseline performance, i.e., some datasets naturally show higher *precision* than others, we evaluated performance in terms of relative rather than absolute metric values. To be specific, for each metric in each dataset, we had 15 values (representing the results from 6 methods and 3 strategies). We computed the median of these 15 values and subtracted it from the original 15 values to obtain the performance gains or losses. By doing so, the results from all datasets can be summarized altogether. Figure 1 exhibits the performance gain or loss in six metrics, and raw scores can be seen in Supplementary Figure S5.

Compared with individual-sample detection, pooled detection improved performance across all evaluation metrics, demonstrating that aggregating information across samples enhances the detectability of rare cell signals. Building on this, applying batch correction prior to pooled detection led to further improvements. Specifically, batch corrected pooled detection achieved higher median values for *Precision*, *Recall*, *F*1-score, *G-mean*, and *MCC* compared with direct pooled detection, indicating improved sensitivity and discriminative power in identifying rare cell populations.

Before discussing the evaluation strategies, we first note that scGPT exhibited consistently poor performance across all three strategies. As its performance levels were similarly low under each strategy, including this method in the strategy comparison would likely bias the overall evaluation. Among the three primary metrics (*Precision*, *Recall*, *F*1), *Precision* values of all methods showed the smallest variation across strategies (Figure 1a). While batch corrected pooled detection achieved the highest median gain, the magnitude of improvement differed by method. scCAD (+0.28) and GapClust (+0.23) showed the largest increases, while CellSIUS (+0.13) and SCISSORS (−0.04) exhibited only minor changes. The only one exception was GiniClust, which displayed a slight increase in *Precision* under direct pooling (+0.11) but a small decrease under batch correction (−0.02). In contrast (to *Precision*), *Recall* exhibited the most pronounced variation among strategies (Figure 1b). Batch-corrected pooled detection substantially increased *Recall* across all methods, with an average gain of +0.11. The largest improvements were observed in GiniClust (+0.41) and SCISSORS (+0.42), followed by a moderate gain for scCAD (+0.32). Notably, SCISSORS and scCAD also performed better under batch correction compared to direct pooling, while GiniClust displayed similar *Recall* levels between the two pooled strategies. By contrast, GapClust showed minimal fluctuation to strategy changes, with comparable results across all three settings. CellSIUS, however, consistently produced negative *Recall* gains (−0.13, −0.28, and −0.09). The *F*1-score, which reflects the balance between *Precision* and *Recall* (Figure 1c), followed a similar pattern to the two previous metrics. Batch-corrected pooled detection yielded the highest overall gains, followed by direct pooling, while individual detection consistently underperformed. Among the five methods, GapClust (+0.24) and scCAD (+0.27) showed the most evident improvements, whereas CellSIUS and SCISSORS exhibited near-zero or slightly negative changes. Overall, the ranking pattern of *F*1-score closely mirrored that of *Recall*, but with smaller variability.

The three comprehensive metrics, *G-mean* (Figure 1d), *Kappa* (Figure 1e), and *MCC* (Figure 1f), which jointly evaluate the balance between sensitivity and specificity, displayed patterns closely mirroring those of *F*1. For *G-mean*, a more pronounced difference was observed across strategies. The batch-corrected pooled detection strategy performed best, achieving the highest values for all methods except GapClust, and showing improvements compared with pooled. In contrast, the individual detection strategy performed the worst, exceeding the median only for GapClust. The impact of strategy changes was particularly evident for GiniClust (−0.07 to +0.24), scCAD (−0.04 to +0.20), and SCISSORS (−0.11 to +0.20). For *Kappa*, method-level differences were more prominent. GapClust achieved consistently higher-than-median results under all strategies, while scCAD showed a substantial improvement from individual to pooled detection (−0.00 to +0.25). GiniClust exceeded the median only under pooled detection, whereas CellSIUS did so only under batch-corrected pooled detection. SCISSORS, however, remained below the median across all strategies. For *MCC*, the best-performing method was scCAD, which showed consistently strong results under both pooled strategies (+0.21 and +0.21). GapClust also performed above the median across all strategies, with further improvement after batch correction (+0.10 to +0.18). GiniClust achieved its best performance under pooled detection, but its score slightly decreased after batch correction (+0.10 to +0.02). In contrast, CellSIUS and SCISSORS remained below the median across all strategies.

**Figure 2:**
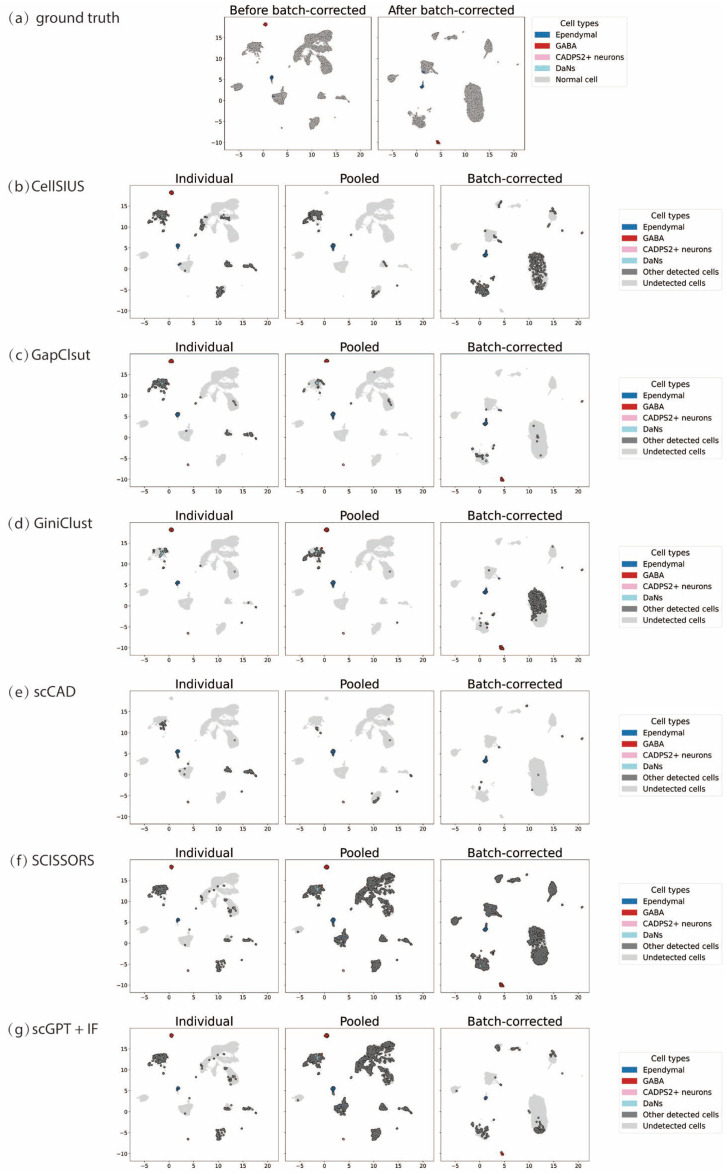
Visualization of rare cell detection results across different analytical strategies and methods on the human midbrain. Gray points represent background cells, while colored points indicate rare cell populations identified by each method. (**a**) UMAP visualization of cells before and after batch correction using ComBat-seq, showing the removal of technical batch effects between samples. (**b**–**g**) UMAP representations of rare cell detection results under three analytical strategies—individual detection, population-level detection, and batch-corrected population-level detection—for five representative methods: (**b**) CellSIUS, (**c**) GapClust, (**d**) GiniClust, (**e**) scCAD, (**f**) SCISSORS, and (**g**) scGPT + IF.

To further illustrate these results, we visualized the Uniform Manifold Approximation and Projection (UMAP) embeddings of the six rare cell detection methods under three analytical strategies (Figure 2). Figure 2 visualizes the detection results of six rare cell type detection methods under three analytical strategies on the Human midbrain dataset. As shown in Figure 2a, batch correction using ComBat-seq effectively reduced inter-sample variation and yielded more coherent rare cell distributions in the feature space, providing a unified reference for pooled analyses. At the method level, GapClust and scCAD exhibited the most accurate and consistent detection results under the batch-corrected pooled strategy. The rare populations detected by these methods were compactly distributed, spatially precise, and showed strong overlap with known rare cell types. Notably, GapClust achieved clear separation among rare subgroups, suggesting that its local density-based modeling becomes more stable after integration, while scCAD prioritized high-confidence, well-defined clusters, demonstrating superior precision. In contrast, CellSIUS and SCISSORS performed less effectively after pooling or batch correction. CellSIUS detected substantially fewer true rare cells, particularly under pooled analysis, where small clusters were merged into larger populations, leading to signal loss. SCISSORS, while achieving higher *recall*, produced dispersed and poorly delineated clusters, indicating that its signal-splitting mechanism is sensitive to residual noise after integration. The UMAP plots for the remaining datasets are shown in Supplementary Figures S6–S8.

Together, these results confirm the quantitative trends: batch-corrected pooled detection substantially improves rare cell identification, yielding more coherent and biologically meaningful rare cell structures. Among all evaluated methods, GapClust, GiniClust, and scCAD demonstrate the highest robustness and reproducibility across multi-sample analyses, making them the most suitable choices for detecting rare cell types in complex and heterogeneous datasets.

### 3.2. Stability Assessment Across Different Rare Cell Proportions

To evaluate the stability and robustness of each method under varying rare cell proportions, we generated simulated datasets derived from real data with rare cell fractions set to 0.5%, 1%, and 1.5%. To be specific, we generated simulated datasets from the real breast cancer data by adjusting the number of cells in the annotated rare population while keeping the total cell number fixed. When a lower rare cell proportion was required, we randomly down-sampled rare cells; when a higher proportion was needed, we oversampled rare cells by duplicating them and adding small random noise to their expression profiles. Non-rare cells were kept unchanged to preserve the original composition and expression variability of the dataset.

**Figure 3:**
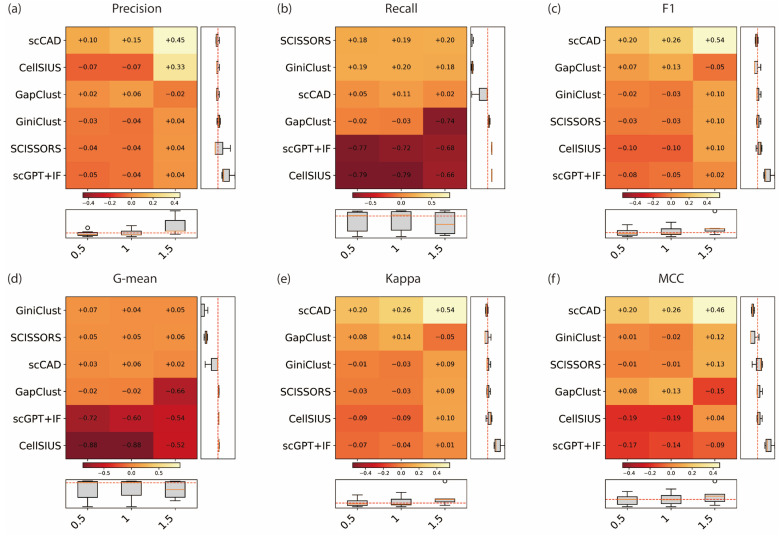
Performance gain/loss of five rare cell detection methods under different rare cell proportions. (**a**) *Precision*, (**b**) *Recall*, and (**c**) *F*1-score, (**d**) *G-mean*, (**e**) *Kappa*, (**f**) *MCC*.

Figure 3 summarizes the gain or loss in six metrics relative to the baseline across different proportions, and raw scores can be seen in Supplementary Figure S6. Overall, the results show that the method’s performance improves as the rare cell proportion increases, except for the *recall*, though the degree of stability varies among methods. Notably, at the highest proportion (1.5%), CellSIUS and GapClust displayed greater fluctuations. This decrease is expected since higher rare cell proportions naturally lead to lower *recall*. scCAD, GiniClust, and SCISSORS exhibited the highest stability across proportions, whereas CellSIUS and GapClust displayed greater fluctuations, with sharp performance declines under low rare cell proportions (0.5%).

For *Precision* (Figure 3a), scCAD again stood out, showing consistently strong gains across all proportions (+0.45, +0.15, +0.10), demonstrating excellent consistency and stability. CellSIUS achieved a marked improvement at 1.5% (+0.33) but decreased slightly at lower proportions (−0.07). GapClust and GiniClust remained relatively stable with small variations (−0.03 to +0.06), while SCISSORS, scGPT+IF showed minimal changes across all conditions (−0.04 to +0.04; −0.05 to +0.04). With respect to *Recall* (Figure 3b), both SCISSORS and GiniClust consistently achieved positive gains across all proportions (approximately +0.18 to +0.20), showing stable performance. scCAD achieved a modest improvement at low proportions (+0.11) but showed a slight decrease at 1.5% (+0.02). GapClust remained close to or below zero (−0.02 to −0.74), while CellSIUS and scGPT exhibited a clear decline across all conditions (−0.79 to −0.66; −0.77 to −0.68). These results indicate that *Recall* generally decreases under extremely low rare cell fractions, except for SCISSORS and GiniClust, which maintained stable detection capability. The *F*1-score results (Figure 3c) mirrored the *Recall* trend. scCAD achieved the greatest and most consistent improvements, with gains of +0.54, +0.26, and +0.20 at 1.5%, 1%, and 0.5%, respectively. GapClust showed mild improvement at 1% (+0.13) but decreased at 1.5% (−0.05) and remained unchanged at 0.5% (+0.07). GiniClust and SCISSORS exhibited similar moderate performance, with *F*1-score gains ranging from −0.03 to +0.10. scGPT+IF also showed this performance, with *F*1-score gains ranging from −0.08 to +0.02. CellSIUS, in contrast, displayed a consistent decline at lower proportions (−0.10 at both 1% and 0.5%), indicating poor stability.

For *MCC*, *G-mean*, and *Kappa* (Figure 3d–f), the results closely paralleled those of *F*1. All three metrics, which jointly evaluate the balance between sensitivity and specificity, demonstrated consistent improvement as the rare cell proportion increased. Batch-corrected pooled detection again delivered the best overall performance across nearly all methods, confirming its robustness under varying signal strengths. Among methods, scCAD achieved the highest and most stable gains (*MCC* +0.46, *G-mean* +0.06, *Kappa* +0.54), indicating its superior ability to maintain classification balance across rare cell proportions. Other methods, such as GiniClust, scGPT+ IF and SCISSORS, exhibited moderate positive changes, while CellSIUS and GapClust tended to decline, particularly under extremely low rare cell fractions. Overall, these comprehensive metrics further support the conclusion that batch-corrected pooling, especially when combined with scCAD, provides the most balanced and reliable rare cell detection performance.

Taken together, results from all performance metrics indicate that scCAD achieved the best overall stability and consistency across rare cell proportions. GiniClust and SCISSORS maintained moderate but persistent positive gains in *Recall* and *F*1-score, showing good robustness. In contrast, CellSIUS and GapClust were more sensitive to changes in rare cell frequency, exhibiting substantial performance drops under low-proportion conditions. Overall, as the proportion of rare cells increases, performance across all methods converges, suggesting that detecting extremely low-frequency rare cells (<1%) remains the most challenging scenario for current methods.

In addition, we examined the ultra-rare scenario in the Human Kidney Atlas dataset, where certain rare cell populations accounted for less than 0.1% of all cells. To evaluate this case, we lowered the rarity threshold accordingly. However, most methods failed to function effectively on this dataset. The failures of CellSIUS and SCISSORS were due to the reasons listed in Supplementary Table S1, while scCAD also failed because it requires dividing the dataset into subclusters smaller than 0.1% and then iteratively merging them. The merging step involving thousands of subclusters took an excessively long runtime, making scCAD unsuitable for such large-scale, fine-grained clustering tasks. In contrast, GapClust, GiniClust3, and scGPT + IF successfully completed the analysis. Among them, both GiniClust3 and GapClust exhibited decreased overall performance compared to the 1% threshold, particularly in *precision*, whereas scGPT + IF remained relatively stable across all evaluation metrics.

### 3.3. Stability with Different Numbers of Rare Cell Types

To evaluate the performance stability of each methods under varying numbers of rare cell types, we constructed three simulation settings in which only 1, 2, or 3 rare cell populations remained below the predefined 1% threshold. Specifically, we derived these settings from real data by selectively retaining or resampling rare populations: (1) pDC was kept as the single rare cell type; (2) both MAST and pDC were used as two rare cell types; and (3) an additional cDC population was included after downsampling its proportion to 1%, resulting in three rare cell populations.

**Figure 4:**
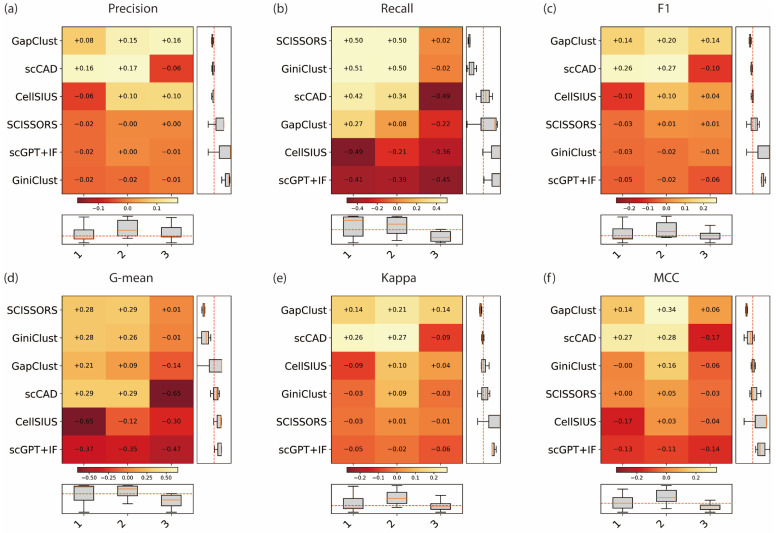
Performance gain/loss of five rare cell detection methods under different numbers of rare cell types. (**a**) *Precision*, (**b**) *Recall*, and (**c**) *F*1-score, (**d**) *G-mean*, (**e**) *Kappa*, (**f**) *MCC*.

Figure 4 summarizes the gain or loss in performance relative to baseline for six metrics across these cell types, and raw scores can be seen in Supplementary Figure S7. Overall, scCAD, GiniClust, and SCISSORS exhibited the most consistent performance across different cell types, while CellSIUS and GapClust showed greater variability, including marked performance drops in certain cell types.

Across the three primary metrics, *Precision* showed generally smaller improvements than *Recall* and *F*1 (Figure 4). GapClust and scCAD achieved steady positive gains across most cell types (+0.16 to +0.17). CellSIUS displayed a slight increase in the two and three types (+0.10) and a decrease in the first type (−0.06). GiniClust, scGPT + IF and SCISSORS showed minimal variation, remaining between −0.02 and +0.00. In summary, while *Precision* remained stable across all cell types, *Recall* and *F*1 exhibited more variability among methods. For *Recall* (Figure 4b), SCISSORS and GiniClust achieved the largest gains, with positive improvements in the first two types (+0.50 to +0.51) and slight decreases in the third type (+0.02 to −0.02). scCAD also showed clear improvement in the first two types (+0.34 and +0.42), followed by a notable decline at the third type (−0.49). GapClust recorded a mild increase in the first type (+0.27), while CellSIUS and scGPT + IF consistently produced negative *Recall* gains across all types (−0.21 to −0.49). As shown in Figure 4c, the *F*1-score displayed a similar pattern to *Precision*. scCAD achieved the highest overall gains in the first two types (+0.26 and +0.27) and a small decrease in the third type (−0.10), maintaining good consistency. GapClust performed moderately (+0.14, +0.20, +0.14), whereas CellSIUS showed a decline in the first type (−0.10). Both SCISSORS and GiniClust remained relatively stable, with small fluctuations (−0.03 to +0.01). scGPT + IF also exhibited a similarly stable pattern, although with slightly lower absolute values (−0.02 to −0.06).

Taken together, the results indicated that scCAD consistently achieved the highest overall performance and stability across different cell types. GiniClust and SCISSORS maintained positive *Recall* gains and cross-type consistency, whereas CellSIUS and GapClust were more sensitive to cell-type differences, showing reduced performance in certain conditions. Overall, variations among cell types suggest that both cellular composition and rarity influence the stability of rare cell detection methods.

### 3.4. Computational Efficiency

As the scale of scRNA-seq datasets continues to expand, computational efficiency has become an important consideration for practical rare cell detection. In this study, we conducted a systematic runtime benchmark across different cell numbers and summarized the measured performance of the six methods under the batch-corrected pooled strategy in Table 3. Note that the reported CPU time corresponds to the sum of CPU time consumed across all active threads, so that the accumulated CPU time across all threads may exceed the elapsed wall time. The results reveal substantial variation in both runtime and memory usage. GapClust and GiniClust were the most computationally efficient, completing detection in approximately 12 min with memory consumption of 31 GB and 47 GB, respectively. In contrast, scCAD and SCISSORS incurred considerably higher computational costs, with peak memory usage reaching 86 GB and 98 GB and wall times of 30 min and 58 min. CellSIUS exhibited the highest resource demand, requiring more than 90 min of computation and over 400 GB of memory, rendering it impractical for large-scale or resource-limited analytical environments.

These trends reflect the underlying algorithmic designs: methods leveraging local density or sparsity-driven heuristics (e.g., GapClust, GiniClust and scGPT + IF) scale more favorably in multi-sample settings, whereas strategies involving iterative modeling, hierarchical refinement, or repeated clustering steps (e.g., scCAD, SCISSORS, and especially CellSIUS) exhibit substantially higher computational burden. Overall, GapClust and GiniClust offer the best trade-off between efficiency and accuracy, while CellSIUS may be unsuitable for large datasets due to its extreme memory footprint.

We further extended the computational efficiency assessment to a large-scale environment (440K cells* × 22K genes). GapClust and GiniClust demonstrated the best scalability, completing the analysis within approximately 30–40 min and consuming 115–198 GB of memory, while remaining fully stable on large HPC nodes. In contrast, scCAD exhibited substantially higher computational cost, requiring 6.5 h of runtime and a peak memory usage of 267 GB, although it successfully completed the task. For scGPT + IF, we further assessed computational performance on the large-scale dataset. While the method completed successfully and identified 4472 rare cells, it required a prolonged runtime of over 40 min and showed non-negligible memory consumption, with a peak usage of approximately 59 GB. SCISSORS showed severe memory inflation when processing the large dataset, with peak memory exceeding 640 GB, and the job was terminated by the system during the silhouette evaluation step, resulting in no valid output. Similarly, CellSIUS failed repeatedly on the large-scale input due to melt data table errors, indicating poor stability under real-world high-volume conditions. For the feasible methods, we conducted visualization analyses (Supplementary Figure S11) and evaluated their performance. scCAD and GapClust exhibited consistently strong performance, with *recall* exceeding 94% and *precision* above 75%, resulting in most other metrics remaining above 85%. GiniClust showed a similar trend to its performance in small sample analyses, achieving nearly perfect *recall* but markedly lower *precision* (21%) compared with scCAD and GapClust. By contrast, scGPT + IF showed substantially inferior performance and identified very few true rare cells.

## 4. Discussion

To investigate the problem of rare cell type detection in multi-sample scRNA-seq, we systematically benchmarked six rare cell detection methods under three analytical strategies—individual detection, pooled detection, and batch-corrected pooled detection. Overall, batch-corrected pooled detection appears to be the most effective strategy, consistently achieving the highest overall performance. Pooled detection also outperformed individual detection but was occasionally hindered by batch effects, which introduced technical noise that confounded biological differences [45]. At the method level, GapClust and scCAD demonstrated the most reliable and stable performance across datasets and strategies. We therefore recommend employing these methods, particularly under batch-corrected pooled detection, as robust and practical tools for rare cell discovery in multi-sample settings.

scGPT + IF represents an initial exploratory attempt to apply single-cell foundation models to rare cell detection. In our current experiments, this method performed sub-optimally, ranking near the bottom across most evaluated metrics. However, we do not interpret this result as evidence that the method itself is fundamentally flawed. Rather, we believe that single-cell foundation models hold substantial promise for advancing rare-cell detection and many other downstream single-cell analytic tasks. The current limitations likely arise from several factors: (1) the pretrained embeddings may not yet be optimized to capture the subtle characteristics of highly imbalanced rare populations; (2) technical variation may still be entangled within the embeddings; (3) the Isolation Forest framework may not fully exploit the nuanced structure encoded in the latent space.

And the potential future improvements include (1) rare-aware pretraining, where foundational models explicitly incorporate rare-population signals during training; (2) graph-informed embeddings or graph-based anomaly detection, enabling better modeling of local neighborhood structures characteristic of rare populations; (3) deep anomaly detection approaches (e.g., autoencoder-based models, energy-based methods, diffusion-based outlier detection) that may capture more complex deviations from common cell states; (4) task-specific finetuning, allowing the pretrained model to better adapt to the unique demands of rare-cell detection. Thus, we view the current results as an early proof of concept rather than a limitation of single-cell foundation models themselves, which are likely to play an increasingly important role in future rare-cell detection frameworks.

At a deeper level, the improved performance of batch-corrected pooled detection in multi-sample scRNA-seq arises from its ability to jointly model cross-sample variability while preserving shared biological structure. Rare cell types, by definition, occupy small fractions within each individual sample, often below the detection threshold for single-sample analyses. When samples are pooled, these rare populations become statistically more visible—but only if technical discrepancies across samples are mitigated. Batch correction plays a critical role here, harmonizing expression spaces so that cells of the same biological identity from different samples can be jointly analyzed. This alignment effectively increases the effective sample size for rare populations and sharpens their separation from background cell types. Methods such as GapClust and scCAD perform particularly well under this framework because their local density–based principles are well-suited to identifying compact, low-frequency clusters that persist across aligned datasets, allowing them to capture consistent rare cell signatures despite inter-sample noise.

Despite these findings, several limitations should be noted. First, while our benchmarking framework accommodates the possibility that rare cell types may appear in only a subset of samples, current detection methods are not explicitly optimized for such heterogeneous occurrence patterns. As a result, rare populations that are condition-specific or sample-restricted may still be under-detected or misclassified when modeled within globally pooled representations. Second, although batch correction improves cross-sample alignment, it introduces a delicate balance between re-moving technical variation and retaining subtle inter-sample biological differences. Over-alignment can obscure genuine sample-specific signals, whereas insufficient correction can inflate false positives by preserving unwanted noise. Third, our evaluations were conducted on datasets with controlled sample sizes and sequencing depths; the scalability, robustness, and interpretability of these methods in larger or more heterogeneous cohorts remain open questions. Future work should focus on adaptive frameworks that integrate sample-level variability into the detection process and ex-tend evaluation to more diverse data contexts, including multi-condition and longitudinal studies.

Accurate detection of rare cell types has important implications for downstream biological analyses. Many rare populations—including plasmacytoid dendritic cells, mast cells, proliferating progenitors, and tissue-resident immune subsets—play disproportionate roles in development, regeneration, and immune regulation. Failure to correctly recover these populations can propagate errors into lineage inference, differential expression analysis, trajectory reconstruction, cell–cell communication modeling, and microenvironment profiling. For instance, omission of proliferative progenitors may distort developmental trajectory estimation, whereas misclassification of rare immune subtypes may obscure key signaling axes in cell–cell communication analyses. The improvements afforded by batch-corrected pooled detection therefore extend beyond computational performance: they enhance the biological interpretability of multi-sample studies by enabling more faithful reconstruction of cellular hierarchies and tissue dynamics.

Although our benchmark is computational, the findings provide a foundation for future biological validation. Rare populations consistently detected under the batch-corrected pooled framework—particularly those showing strong expression of canonical marker genes—represent candidates for experimental confirmation. Techniques such as flow cytometry, immunohistochemistry, RNA in situ hybridization, and lineage tracing could be used to validate the molecular identity, functional relevance, and spatial distribution of predicted rare subsets. Moreover, perturbation studies or condition-specific experiments may help determine whether these rare populations possess regulatory or disease-associated roles. Integrating such orthogonal evidence would further strengthen the translational significance of rare-cell detection in multi-sample scRNA-seq.

Finally, we note that the benchmark relies on abundance- and annotation-based definitions of rare cell types, which, while practical and reproducible, do not constitute a biological gold standard. As such, the reported performance should be interpreted in light of this limitation, and future work incorporating biological or orthogonal validation will further strengthen benchmarking reliability. 

## 5. Conclusions

In this study, we conducted a comprehensive benchmark for the general strategy and method selection for rare cell type detection in scRNA-seq. Our results demonstrate that the batch-corrected pooling detection strategy consistently yields the most accurate and stable performance, while scCAD emerges as the most robust and generalizable method. This study provides the strategy-level comparison of rare cell type detection under multi-sample settings and highlights the importance of batch correction and method selection in achieving accurate and reproducible rare cell type identification. With the increasing popularity of multi-sample scRNA-seq experiments, the presented work offers a foundation for future methodological development and guides researchers in applying reliable approaches for rare cell type detection in large-scale single-cell studies.

## Figures and Tables

**Table 1:** Rare cell type detection methods.

Method	Key Principle	Requires Clustering	Outlier Score	Platform
CellSIUS	Bimodal gene expression in clusters	yes	no	R
GapClust	Local density and expression gap	no	no	R
GiniClust	High-Gini gene feature + clustering	no	no	Python
scCAD	Clusters decomposition + anomaly	yes	yes	Python
SCISSORS	Silhouette-based subclustering	yes	no	R
scGPT + IF	Embedding and isolation forest	no	yes	Python

**Table 2:** Datasets of rare cell detection.

Datasets	Cells	Genes	Types	Rare Cells	Proportion	Samples	Data Source
Breast cancer	57,411	22,815	12	522	0.9%	10	GEO GSE266919
Human midbrain	41,435	26,737	12	1265	3%	11	GEO GSE157783
Mouse hippocampus	29,519	27,998	8	114	0.4%	13	www.mousebrain.org
Human Kidney atlas	87,647	29,394	26	1689	1.9%	11	GEO GSE183279

**Table 3:** Computational Efficiency on Breast Cancer.

Method	Wall Time (min)	CPU Time (s)	Max RSS (GB)	Notes
CellSIUS	90.8	130,099	407	Extremely high memory usage; slowest runtime
GapClust	11.9	972	31.1	fast; lowest memory consumption
GiniClust	11.5	2491	46.5	Fastest runtime; moderately high memory usage
scCAD	57.9	15,681	97.8	High computational cost and high memory usage
SCISSORS	29.5	1768	85.7	Moderate runtime but still requires substantial memory
scGPT + IF	12.1	929	31.1	fast; lowest memory consumption

## Data Availability

The datasets analyzed in this study are publicly available from the Gene Expression Omnibus (GEO) database under accession numbers GSE266919, GSE157783, and GSE183279 and www.mousebrain.org.

## Supplementary Materials

The following supporting information can be downloaded at: https://www.mdpi.com/article/10.3390/genes17010031/s1, Figure S1: marker genes on breast cancer across batch-correction; Figure S2: marker genes on mouse hippocampus across batch-correction; Figure S3: marker genes on human midbrain across batch-correction; Figure S4: marker genes on human kidney atlas across batch-correction; Figure S5: Performance raw of five rare cell detection methods under three analytical approaches across all datasets; Figure S6: Visualization of rare cell detection results on breast cancer; Figure S7: Visualization of rare cell detection results on mouse hippocampus; Figure S8: Visualization of rare cell detection results on human kidney atlas; Figure S9: Performance raw of six rare cell detection methods under different rare cell proportions; Figure S10: Performance raw of six rare cell detection methods under different rare cell types; Figure S11: Visualization of rare cell detection results across different analytical strategies and methods on human midbrain; Table S1: failed detection.

## Abbreviations

The following abbreviations are used in this manuscript:

Bfoc	Follicular B cell
cDC	Conventional dendritic cell
FN	False negatives
FP	False positives
IF	Isolation Forest
PCA	Principal component analysis
pDC	Plasmacytoid dendritic cell
scRNA-seq	Single-cell RNA sequencing
TN	True negatives
TP	True positives
UMAP	Uniform Manifold Approximation and Projection
