# Supplementary material for: Revealing the Best Strategies for Rare Cell Type Detection in Multi-Sample Single-Cell Datasets
Source: Genes (Basel). 2025 Dec 29;17(1):31. doi: 10.3390/genes17010031 (PMC12840603; doi:10.3390/genes17010031)
Supplement: Supplementary file 1 [file genes-17-00031-s001.zip › genes-4066932-supplementary.pdf]

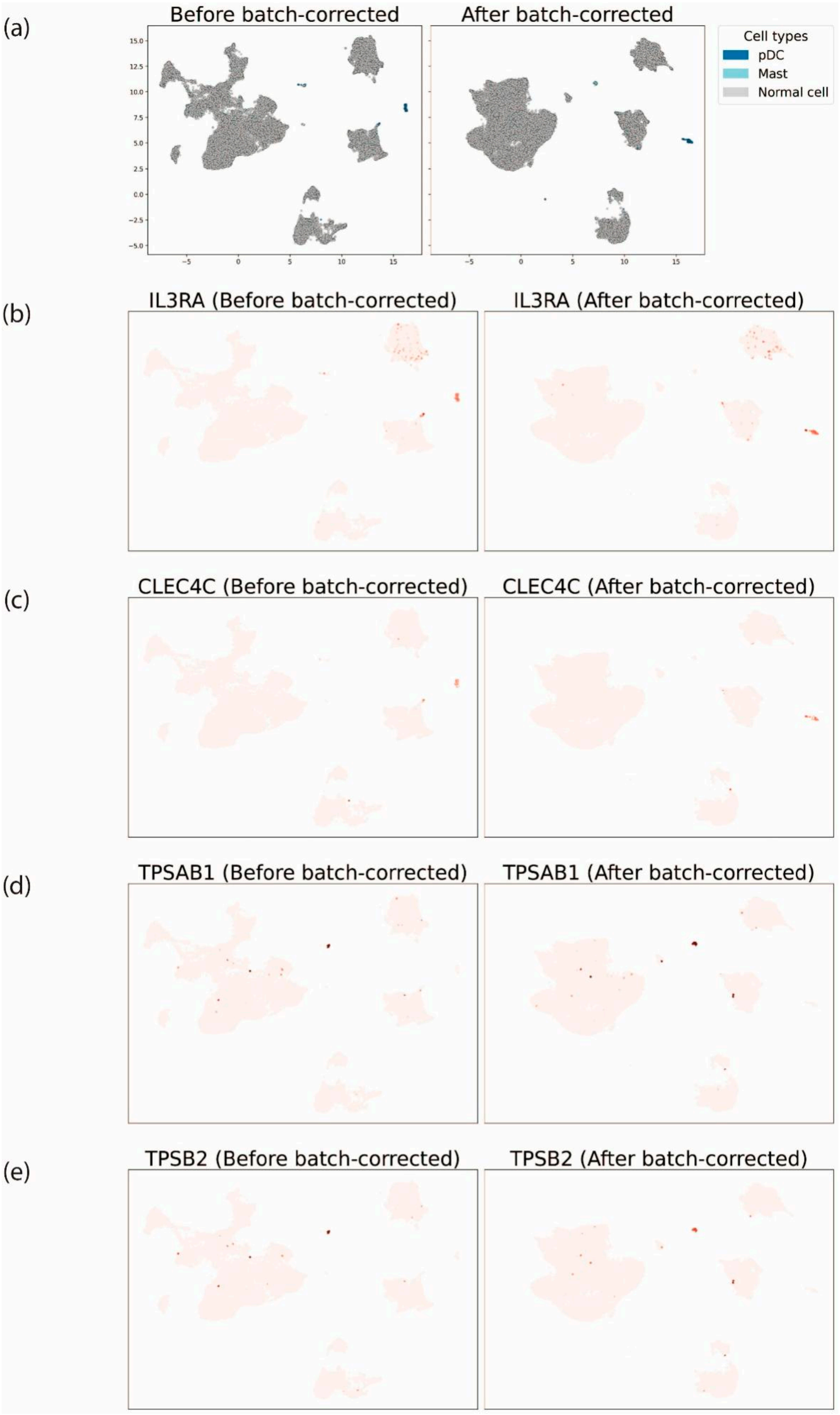

Figure S1. marker genes on breast cancer across batch-correction. (a) Rare cell, (b) IL3RA, (c) CLEC4C, (d) TPSAB1, (e) TPSB2.

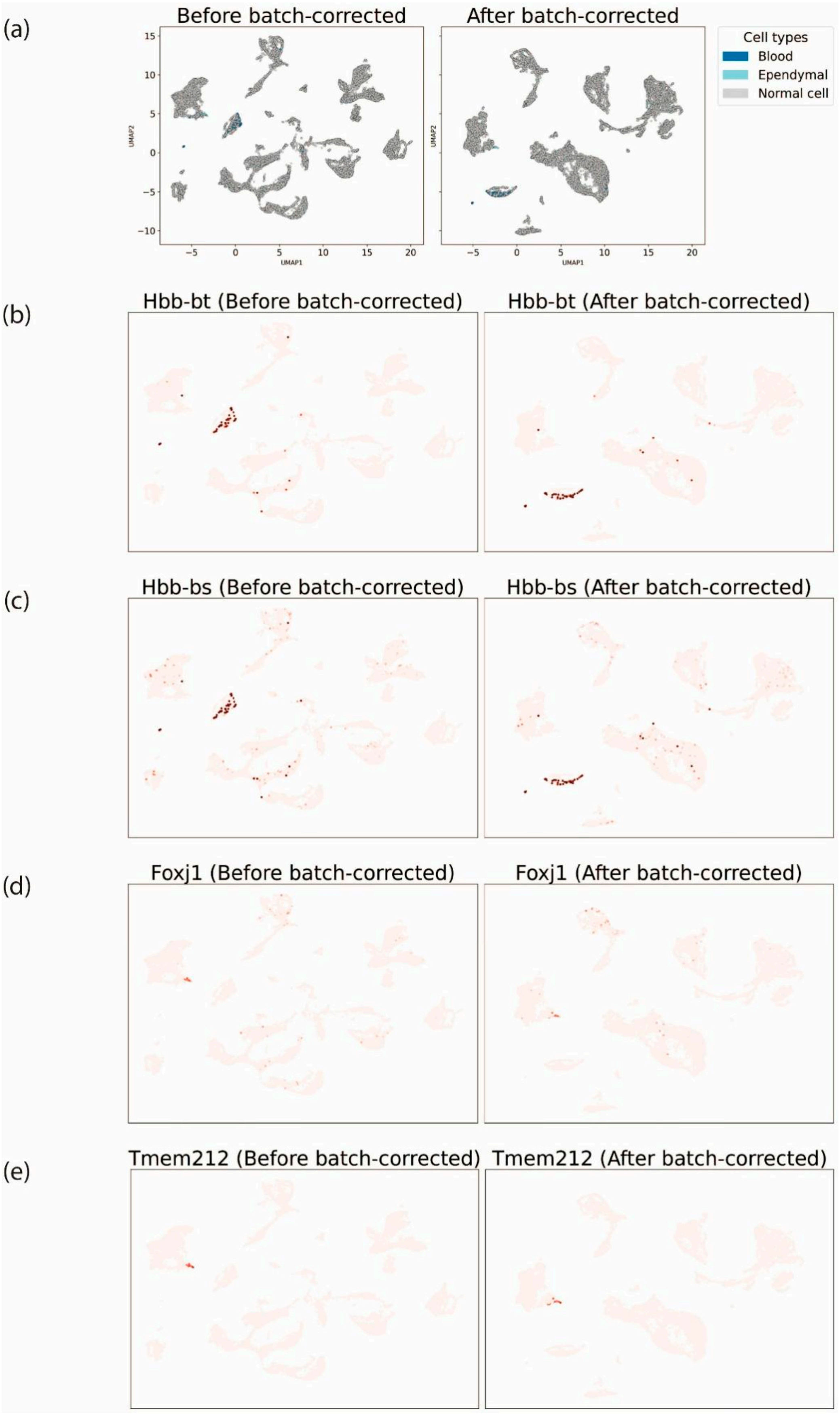

Figure S2. marker genes on mouse hippocampus across batch-correction. (a) Rare cell, (b) Hbb-bt, (c) Hbb-bs, (d) Foxj1, (e) Tmem212.

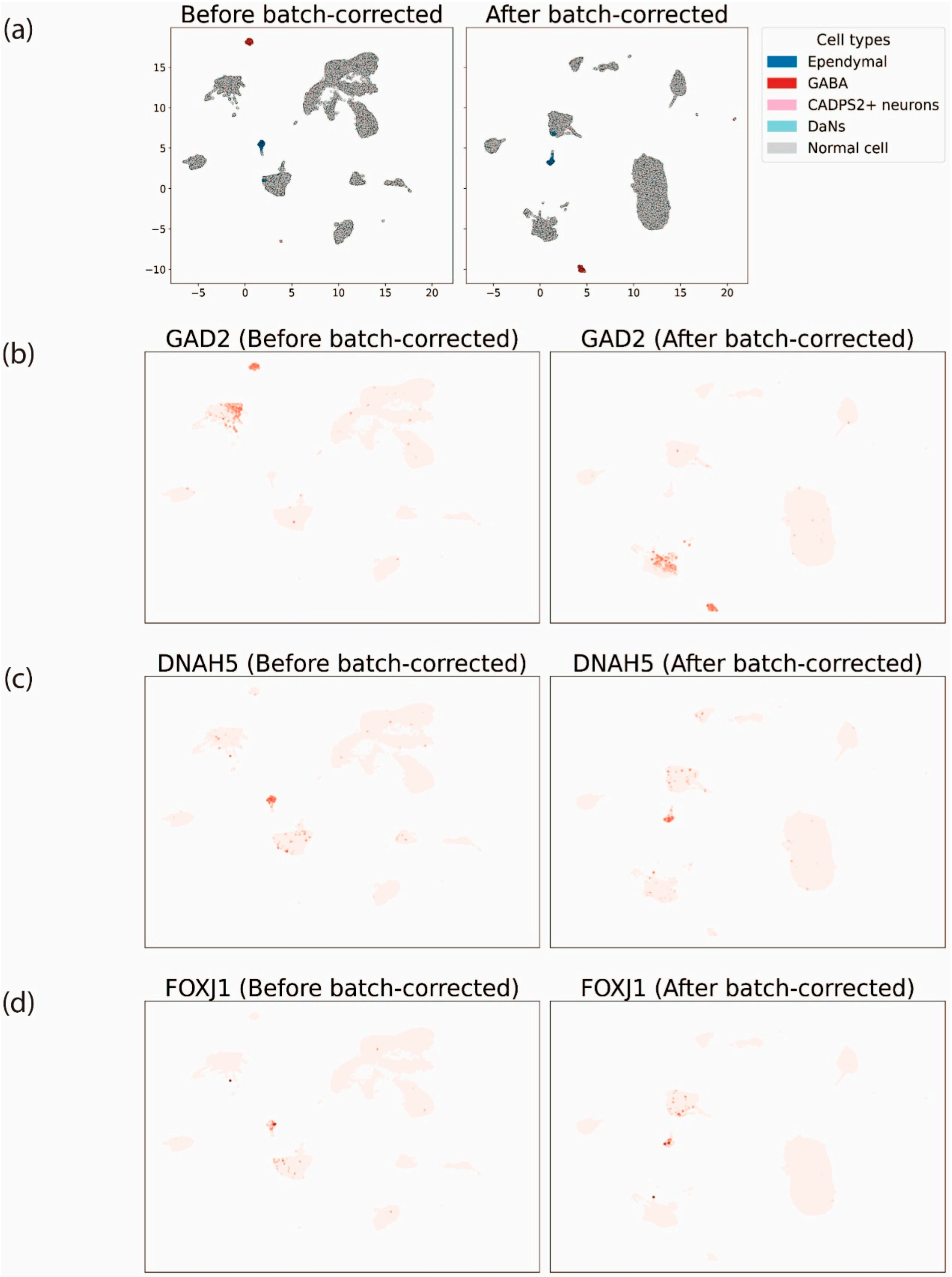

Figure S3. marker genes on human midbrain across batch-correction. (a) Rare cell, (b) GAD2, (c) DNAH5, (d) FOXJ1.

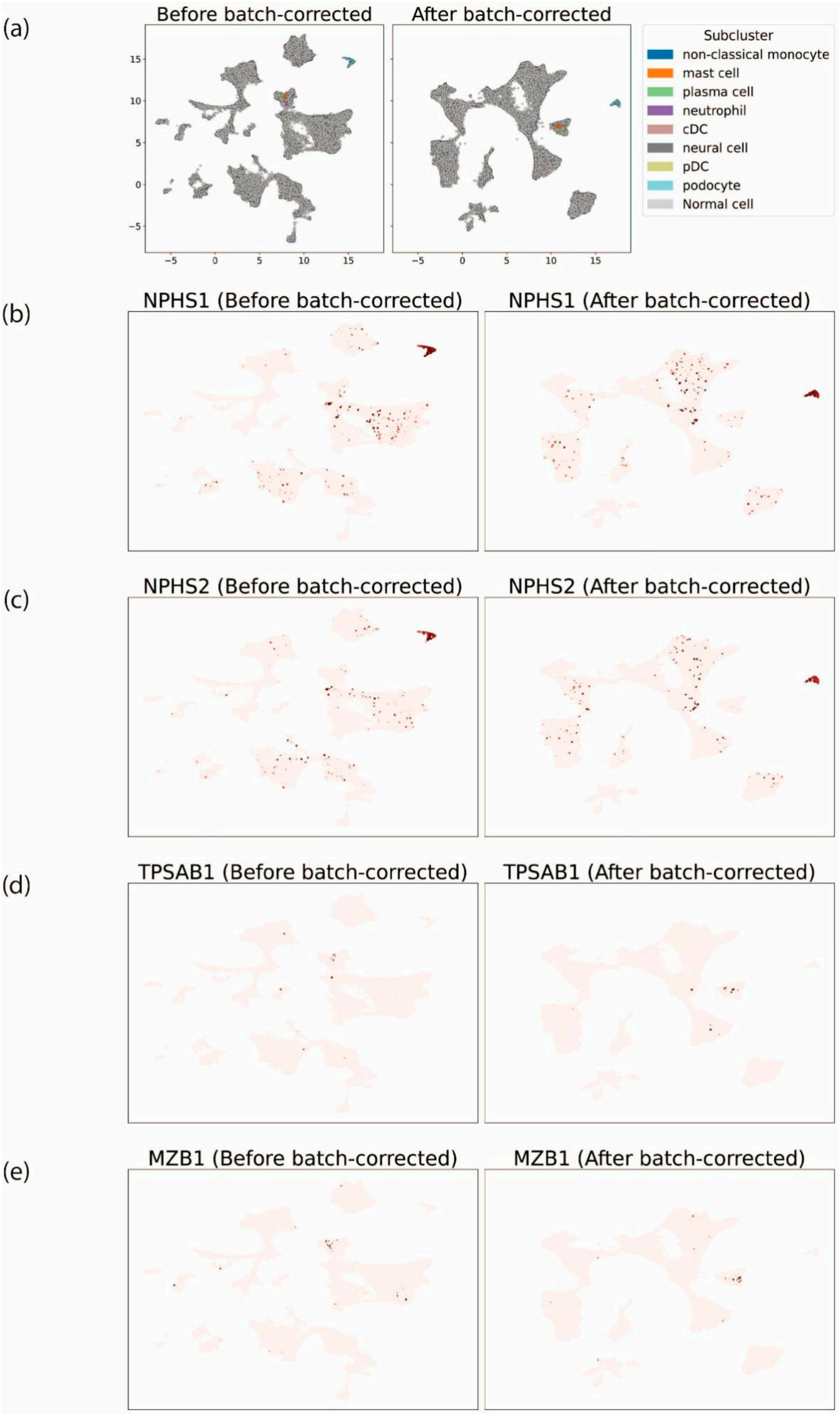

Figure S4. marker genes on human kidney atlas across batch-correction. (a) Rare cell, (b) NPHS1, (c) NPSH2, (d) TPSAB1, (e) MZB1.

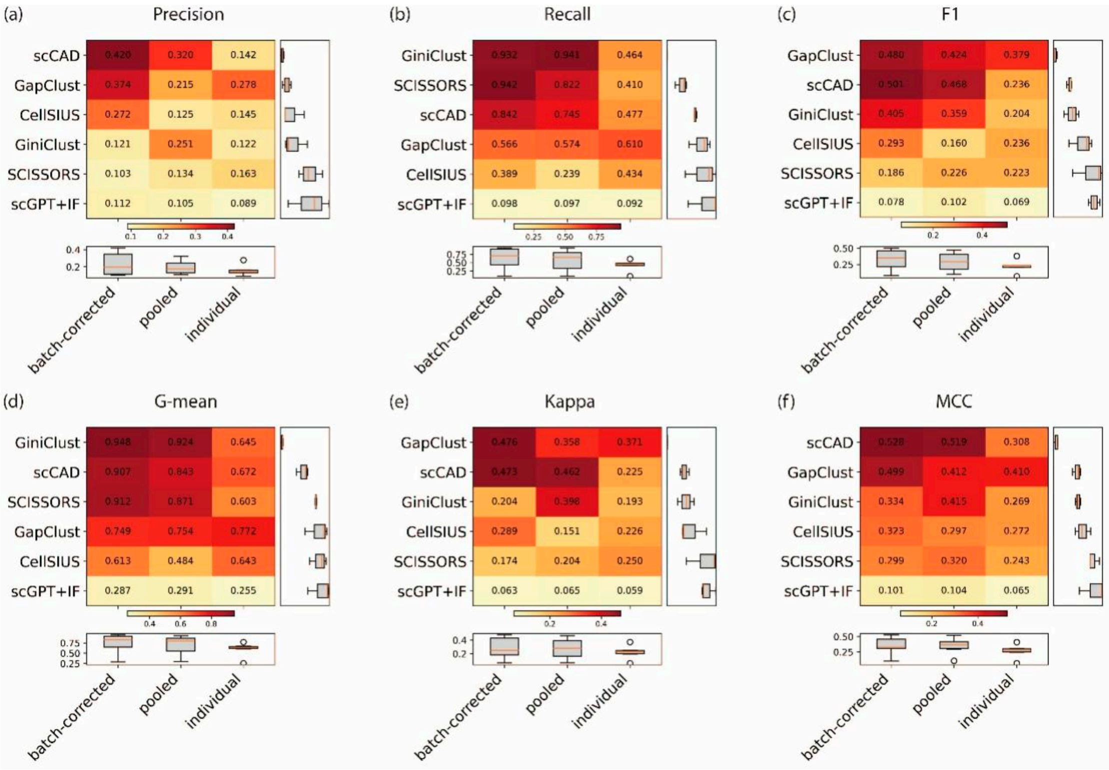

Figure S5. Performance raw of six rare cell detection methods under three analytical strategies across all datasets. (a) Precision, (b) Recall, and (c) F1-score, (d) G-mean, (e) Kappa, (f) MCC.

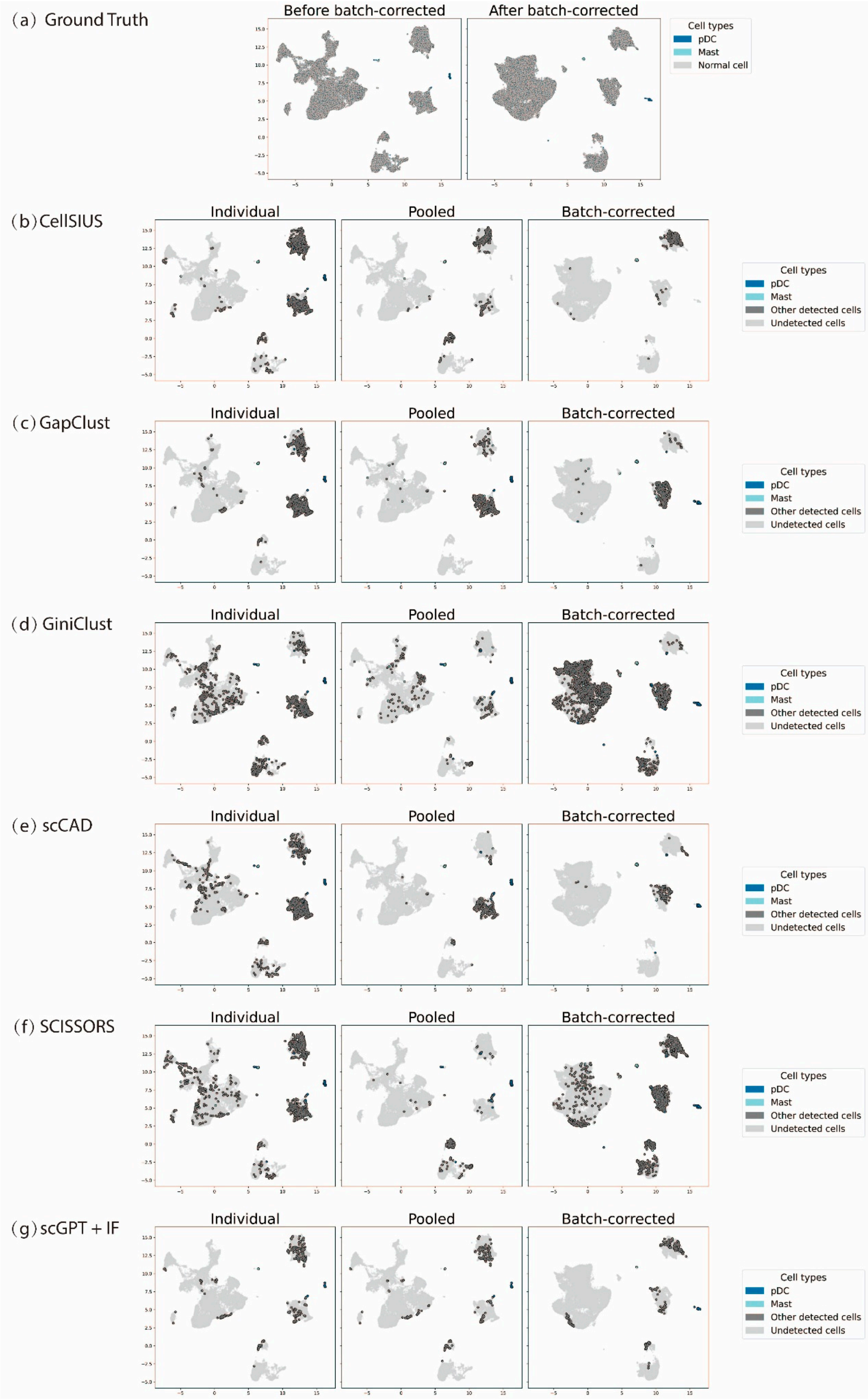

Figure S6. Visualization of rare cell detection results across different analytical strategies and methods on

breast cancer. Gray points represent background cells, while colored points indicate rare cell populations identified by each method. (a) UMAP visualization of cells before and after batch correction using ComBat-seq, showing the removal of technical batch effects between samples. (b–g) UMAP representations of rare cell detection results under three analytical strategies—individual detection, population-level detection, and batch-corrected population-level detection—for five representative methods: (b) CellSIUS, (c) GapClust, (d) GiniClust, (e) scCAD, (f) SCISSORS, and (g) scGPT + IF.

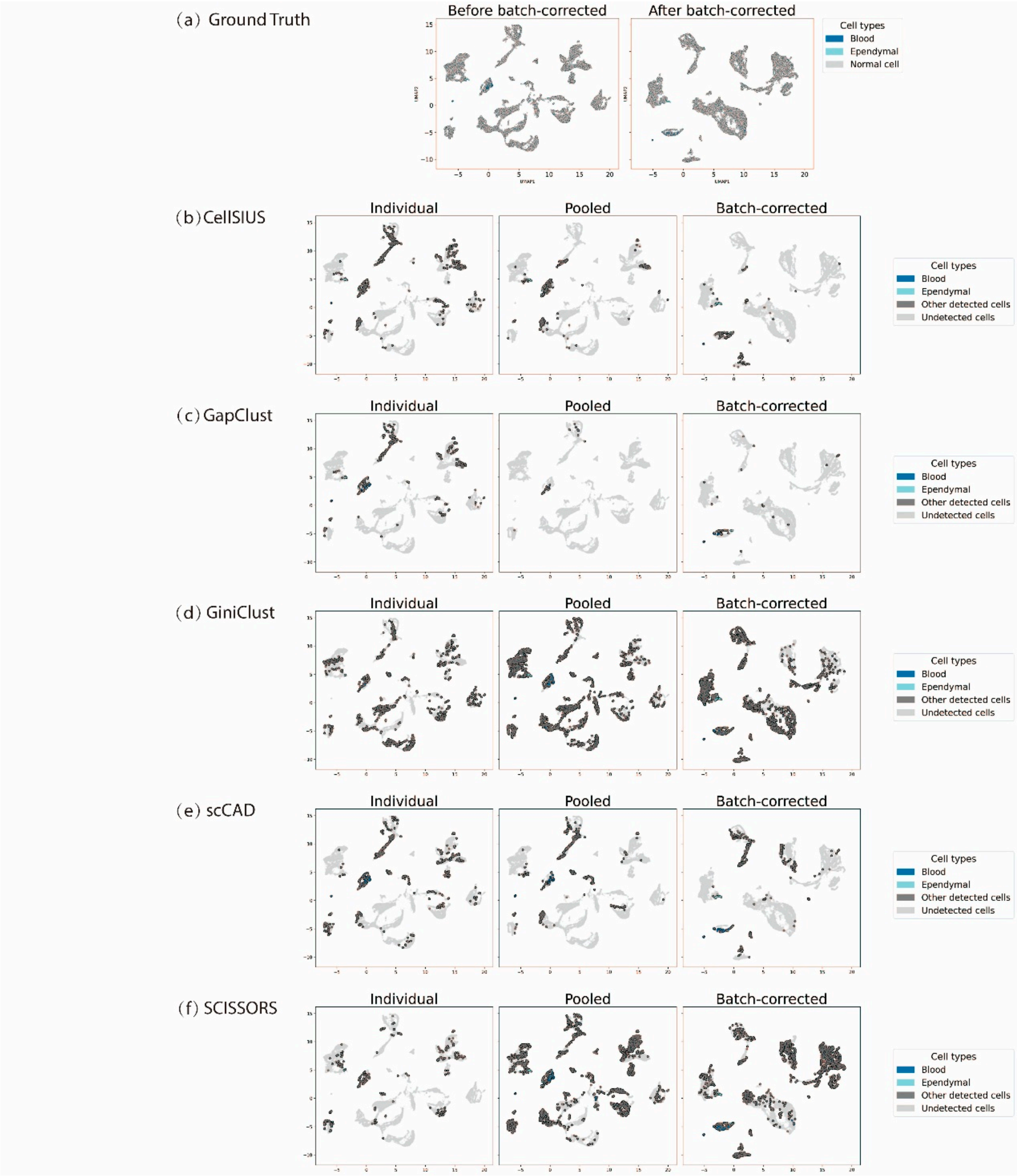

Figure S7. Visualization of rare cell detection results across different analytical strategies and methods on mouse hippocampus. Gray points represent background cells, while colored points indicate rare cell populations identified by each method. (a) UMAP visualization of cells before and after batch correction using ComBat-seq, showing the removal of technical batch effects between samples. (b–f) UMAP representations of rare cell detection results under three analytical strategies—individual detection,

population-level detection, and batch-corrected population-level detection—for five representative methods: (b) CellSIUS, (c) GapClust, (d) GiniClust, (e) scCAD, and (f) SCISSORS.

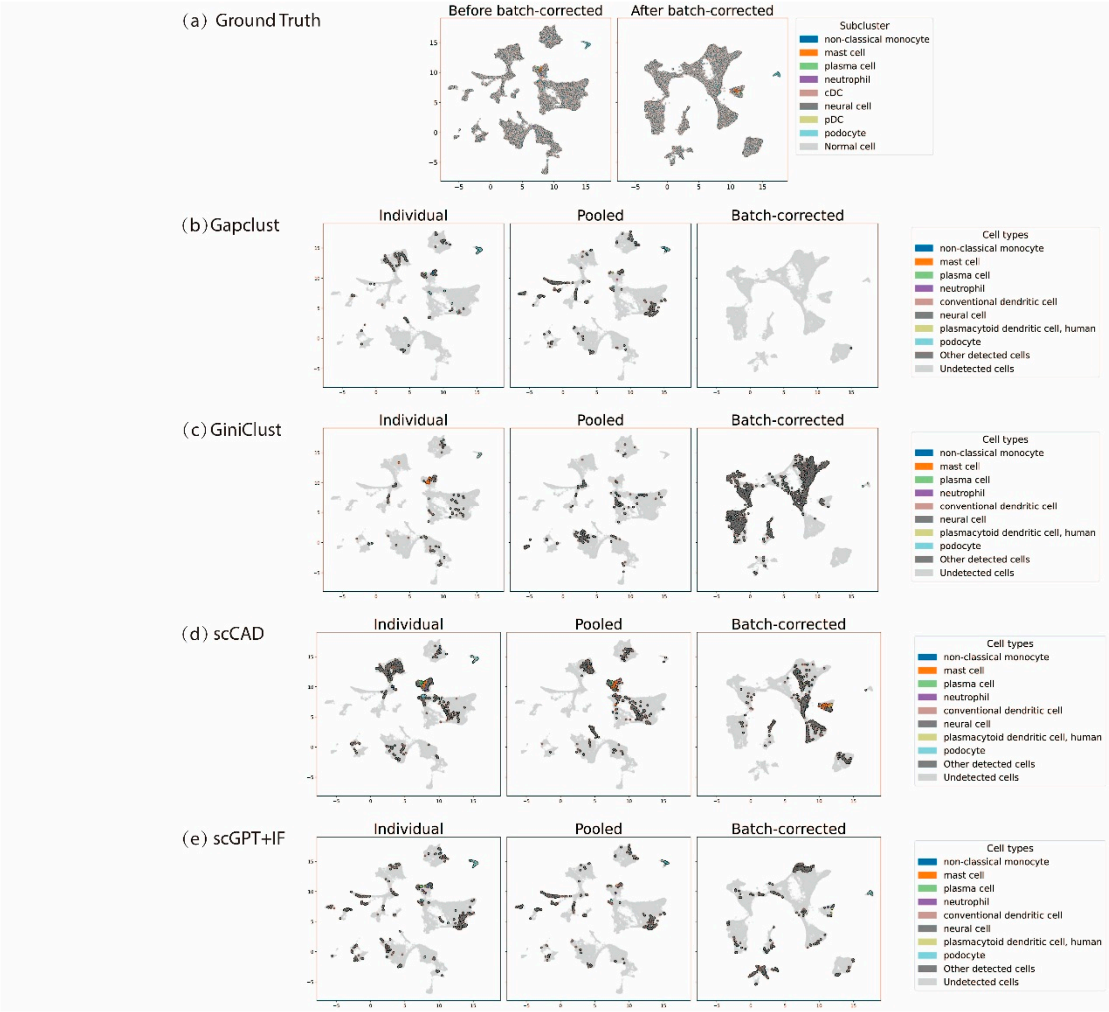

Figure S8. Visualization of rare cell detection results across different analytical strategies and methods on mouse hippocampus. Gray points represent background cells, while colored points indicate rare cell populations identified by each method. (a) UMAP visualization of cells before and after batch correction using ComBat-seq, showing the removal of technical batch effects between samples. (b–e) UMAP representations of rare cell detection results under three analytical strategies—individual detection, population-level detection, and batch-corrected population-level detection—for five representative methods: (b) GapClust, (c) GiniClust, (d) scCAD, and (e) scGPT + IF.

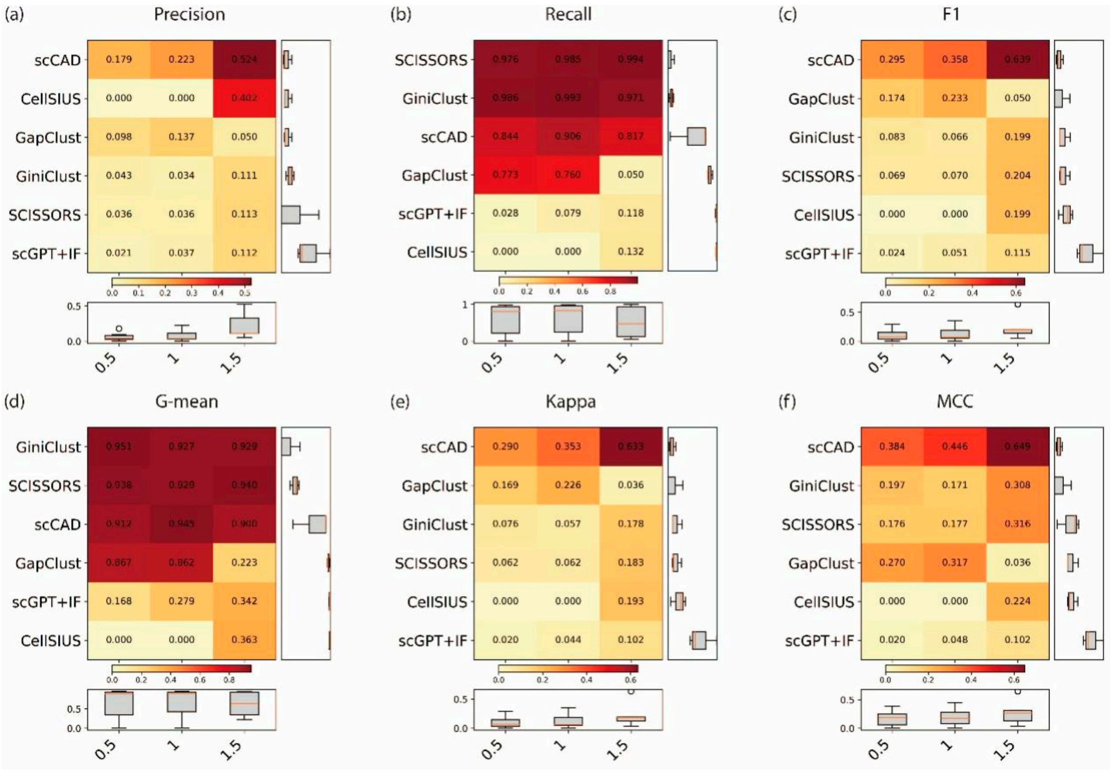

Figure S9. Performance raw of six rare cell detection methods under different rare cell proportions. (a) Precision, (b) Recall, and (c) F1-score, (d)G-mean, (e) Kappa, (f) MCC.

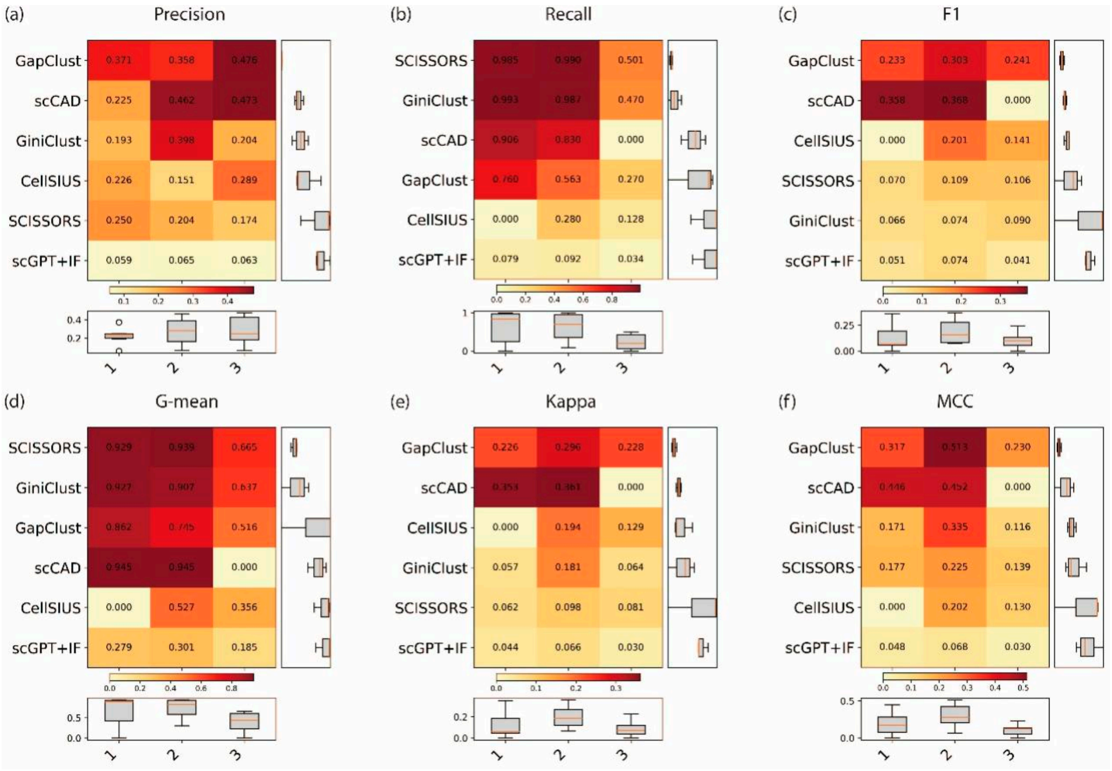

Figure S10. Performance raw of six rare cell detection methods under different rare cell proportions. (a) Precision, (b) Recall, and (c) F1-score, (d)G-mean, (e) Kappa, (f) MCC.

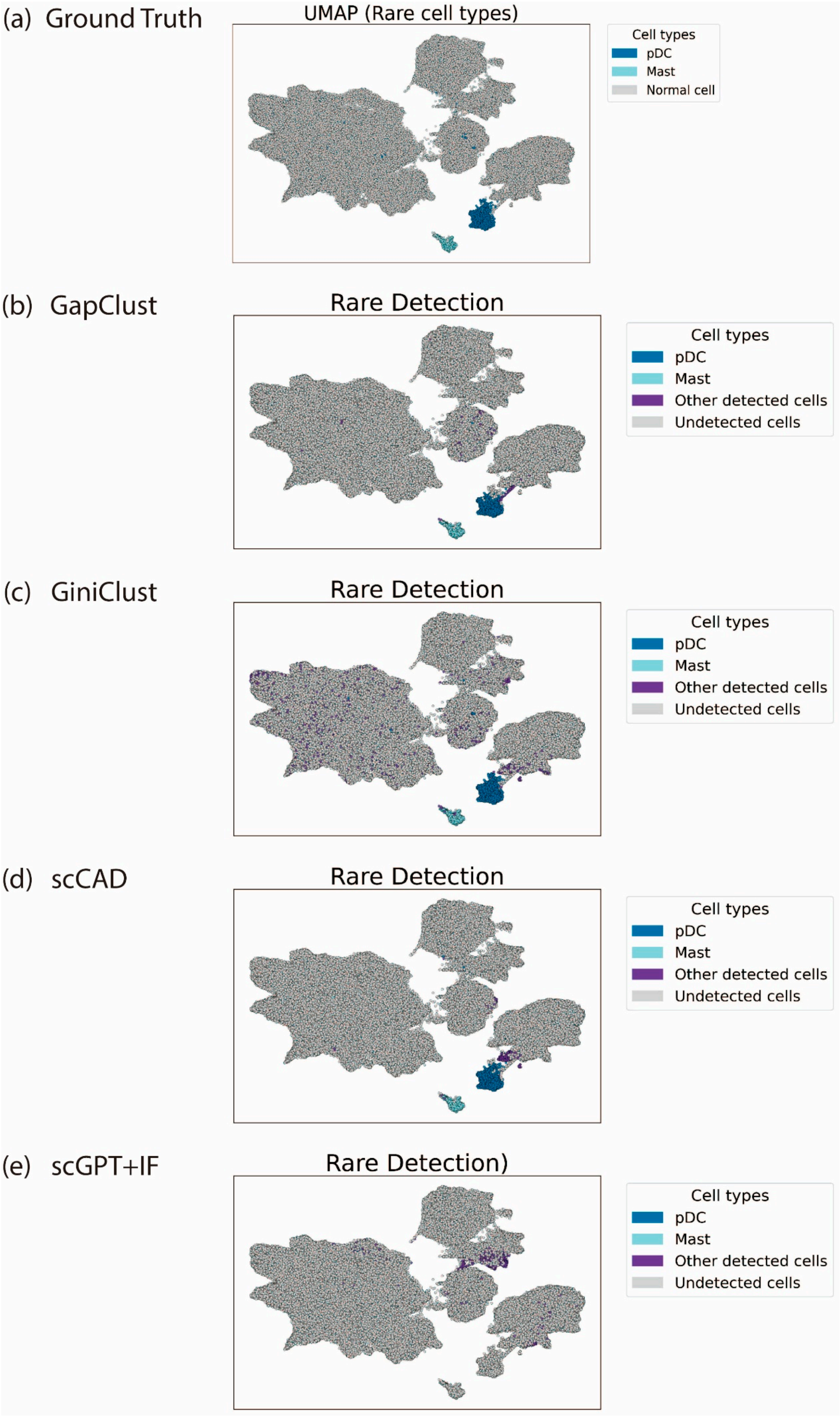
(a) Ground Truth
UMAP (Rare cell types)
Cell types
pDC
Mast
Normal cell
(b) GapClust
Rare Detection
Cell types
pDC
Mast
Other detected cells
Undetected cells
(c) GiniClust
Rare Detection
Cell types
pDC
Mast
Other detected cells
Undetected cells
(d) scCAD
Rare Detection
Cell types
pDC
Mast
Other detected cells
Undetected cells
(e) scGPT+IF
Rare Detection)
Cell types
pDC
Mast
Other detected cells
Undetected cells

Figure S11. Visualization of rare cell detection results across different analytical strategies and methods on human midbrain. Gray points represent background cells, while colored points indicate rare cell populations identified by each method. (a) UMAP visualization of cells before and after batch correction using ComBat-seq, showing the removal of technical batch effects between samples. (b–e) UMAP representations of rare cell detection results under three analytical strategies—individual detection, population-level detection, and batch-corrected population-level detection—for five representative methods: (b) GapClust, (c) GiniClust, (d) scCAD, and (e) scGPT + IF.

Table S1. failed methods and reasons

Method	Datasets	Error type	Analysis	Strategies
CellSIUS	Human Kidney atlas	Error in melt. data. table	The internal call to data. table::melt() in CellSIUS caused memory overflow when processing large matrices.	Batch-corrected
SCISSORS	Human Kidney atlas	Error in silhouette.default	The ComputeSilhouetteScores() function cannot handle vectors exceeding 2^{31}−1 elements (R internal memory limit)	Batch-corrected
CellSIUS	Human Kidney atlas	Error in melt. data. table	The internal call to data. table::melt() in CellSIUS caused memory overflow when processing large matrices	Pooled
